# User knowledge factors that hinder the design of new home healthcare devices: investigating thirty-eight devices and their manufacturers

**DOI:** 10.1186/s12911-021-01464-3

**Published:** 2021-05-21

**Authors:** Fan Yang, Abdullah Al Mahmud, Tao Wang

**Affiliations:** 1 Guangzhou Academy of Fine Arts, Guangzhou, China; 2 Swinburne University of Technology, Melbourne, Australia

**Keywords:** Product design, User performance, Usability, Home health care device, Medical devices and technologies

## Abstract

**Background:**

The demand for home healthcare devices arises; however, many home healthcare devices on the market are not designed to reflect the needs and features of the end-users. This study explored the user knowledge factors that hindered the design of new home healthcare devices and the interrelationships between the factors.

**Methods:**

The abovementioned factors were identified from analysing the project documents of thirty-eight carefully selected home healthcare devices produced by five manufacturers; followed by interviewing the thirty stakeholders playing key roles in developing the devices.

**Results:**

The design of the home healthcare devices was influenced by (1) the user insights utilised in formulating project strategies; (2) the sources of user information; (3) the execution of user research; and (4) the formulation of the manufacturers’ principal innovation processes.

**Conclusions:**

The users’ characteristics and needs were not sufficiently reflected in developing new home healthcare devices. One root cause was that the end-users were not perceived by the manufacturers as a key success factor in most cases, given that most of the devices were initiated following the public sector’s requests. Actual or potential applications of this study include the facilitation of the appropriate application of human factors methods in developing new home healthcare devices and the improvement of the user performance of the end-devices.

**Supplementary Information:**

The online version contains supplementary material available at 10.1186/s12911-021-01464-3.

## Background

Traditionally home healthcare services have been performed by medical practitioners such as midwives, carers and travelling doctors visiting people’s homes. As opposed to the short visits generally aimed at meeting specific needs in the past, modern home health care needs to address on-going and long-term medical and health requirements because of the global trend of a rapidly aging population, and rising prevalence of lifestyle-related chronic diseases [36]. Modern home health care is also expected to provide both care receivers and their families greater comfort, pleasure, and wellbeing, beyond problem-oriented “one-off” solutions that traditionally focus only on clinical requirements [53]. These challenges are intensified by the increasingly scarce human, capital, and operational resources in many countries [23, 49]. In this context, there is increasingly widespread use of medical and healthcare devices to deliver home healthcare services [40]. According to the US Food and Drug Administration (FDA), Home Healthcare Devices (HHCDs) refer to “medical devices intended for users in any environment outside of a professional healthcare facility”, including “devices intended for use in both professional healthcare facilities and homes” [18]. HHCDs accommodate a wide range of equipment and systems from managing chronic diseases at one end of the spectrum, to preventing diseases at the other. They include simple thermometers to complex equipment like oxygen generators and home dialysis machines.

Promoting the use of HHCDs can improve the users’ wellbeing, such as improved independence and confidence in life, beyond the physiological parameters of disease control [54]. It can also mitigate the current pressure on the healthcare system. Obtaining these benefits is dependent upon correct and regular use of the devices [34]. However, user safety incidents involving healthcare devices occur every day and have become a common source of patient injury and death. For example, the research from Zhang et al. [56] shows that in many cases medical devices have user interfaces that are so poorly designed and difficult to use that they invite a variety of human errors. Other research suggests that injuries resulting from medical device use errors far exceeds injuries arising from device failures [8]. Compared with medical devices intended to be used within formal hospital settings, HHCDs are more frequently used under unsuitable conditions [18]. The resulting complexity of the methods of using HHCDs has led to a parallel intensification of the risks related to bad functioning and/or failure to function or misunderstanding how to use the interface, control and adjustment elements, assembly sequences, activation, etc. [50]. In this context, the designing of HHCDs needs to take human factors including safety, ease of use, and people’s subjective wellbeing into special consideration, for example, how to correctly plan the visibility and legibility of information, how to manage manual controls, and how to simplify the reading and to interpret the digital interface.

Via the deployment of User-Centred Design (UCD) principles and methods, focusing on human factors has been broadly recognised and promoted by academic literature as a central principle for new healthcare device development (e.g., [5, 7, 34, 44]. To push the enforcement of human factors engineering methods within the development processes of medical devices, international standards bodies have established various standards and regulations to which the manufacturers have been obliged to adhere. One important international standard is IEC 60601-1 [25] that has introduced the general requirements for basic safety and essential performance of medical electrical equipment. This is classified further in IEC 60601-1-11 [26] that applies to the performance of medical electrical equipment and systems for use particularly, in the home healthcare environment. On the other hand, IEC 62366-1 [27] extends the requirement of incorporating human factors engineering methods to the development of all medical devices and systems, not just electrical devices. It specifies a process for a manufacturer to analyse, specify, develop and evaluate the usability of a medical device as it relates to safety. In early 2010, IEC 62366 was harmonised by the EU Medical Device Directive meaning that it is now a legal requirement for medical device manufacturers to formally address the usability of a device before placing it on the market anywhere in Europe.

Despite the abovementioned efforts, the design of many existing HHCDs, even of those produced by leading companies, fail to reflect critical needs and requirements of the end-users [55]. In England, for example, the National Health Services England (NHS [41] received over forty-thousand reports of patient safety incidents involving healthcare devices in 2013, with the exact number likely to be higher due to reporting and coding issues. Many of these failures are not due to flawed technology, but rather due to the lack of systematic considerations of human issues, during the design stage and the implementation stage of a new device [43]. The existing studies have revealed some causes of this dilemma, for example, Money et al. [39] and Martin et al. [38] pointed out that healthcare device manufacturers often avoid employing formal UCD/human factor engineering methods, due to a shortage of resources and the perception that such methods are often too resource-intensive. Moreover, the culture of training people to adapt to poorly designed technology, rather than designing technology to fit people's characteristics [43]. However, these conclusions were drawn from the studies upon medical devices as a whole, that did not take full account of the specificities of the HHCD sector.

Addressing these concerns, this study aimed to clarify the critical user knowledge factors that hindered the design of effective HHCDs; and determine the interrelationships between the factors.

## Methods

In investigating the research targets, we adopted a mix of qualitative and quantitative methods, with reference to Wisdom and Creswell [52], Mahmud and Martens (2015), and Kumar and Wallace [30]. According to the Agency for Healthcare Research and Quality (AHRQ) in the US [52] and some scholars (e.g., [24, 42], mixed methods (qualitative and quantitative) can be an ideal technique to assess complex interventions in the home healthcare sector.

### Materials and participants

According to Flyvbjerg [19], case studies are necessary to understand a phenomenon to any degree of thoroughness, while statistical studies are necessary to understand the prevalence of a phenomenon. This study was conducted on thirty-eight HHCDs and their manufacturers (Table 1. In terms of functionality, these devices can be categorised into four types: (1 assistive technologies (n = 13, including three crutches, two hearing aids, six orthotics and two wheelchairs; (2 meters and monitors (n = 6, including four blood glucose meters and two electrocardiogram monitors; (3 respiratory equipment (n = 4, including one forced airway devices, one oxygen, and two suction; (4 telehealth and telecare equipment (n = 15; including three-bed occupancy sensors, one bogus caller, one chair absence sensors, one epilepsy sensors, one enuresis sensors, five fall detectors, one monoxide alarms, and two pressure mats. They covered most types of HHCDs on the market. These devices were produced by five manufactures that are hereafter referred to as M1, M2, M3, M4 and M5. M1 was a large company and was a market leader. M2-M5 were Small and Medium-Sized Enterprises (SMEs, given that the HHCD sector was dominated by SMEs (European [17, 45]. For reasons of confidentiality agreements, this paper will not name or otherwise identify these devices and their manufacturers,they remain anonymous by the use of pseudonyms.

**Table 1:** Research participants and activities

Interviews (Sum = 30, total duration≈ 35 h)	Individuals consulted (Sum = 18)	Organisations (n = 5)	Number of HHCDs (Sum = 38)
	Key informants (n = 12)		
Two face-to-face interviews with each respondent (Duration = 1–1.5 h)	Five product managers, Two project managers	Manufacturer 1 (M1)	26
	Two project managers	M 2	3
	One project manager	M 3	3
	One project manager	M 4	3
	One project manager	M 5	3
	Other informants (n = 6)		
One face-to-face interview with each respondent (Duration = 1–1.5 h)	One quality director, One R&D manager, One service manager, One innovation director, Two service and installation engineers	M 1	

We initially selected fifty HHCDs as appropriate research targets and then contacted the nine manufacturers produced the devices. Later on, M1-M5 agreed to provide the information concerning the development of thirty-eight HHCDs on the preliminary list. Taking into account most of the participants’ intentions as well as our own purposes in the study, we firstly investigated three HHCDs recently produced by each of the five manufacturers, through interviewing the devices’ project leaders (1.5–2 h each) and assessing the project documents that the project leaders provided. Further investigation was conducted at M1. Its chief technology officer consented to the first author’s access to the project information of an extensive portfolio of M1′s products (n = 26); and the staff members representing all of the key departments involved in M’s New Product Development (NPD) processes. These informants included seven project leaders (five product managers and two project managers), the quality director, the R&D manager, the service manager, and two service and installation engineers (see Table 1). We were allowed to perform a more in-depth investigation within M1 since the first author had established deeper cooperation with the company before this study. All of the interviews were conducted in person by the first author who had over ten years’ research and product design experiences at universities, large international companies and SMEs.

The study was undertaken following the University of the Arts London's Code of Practice on Research Ethics and was submitted to and approved by the university.

### Procedure and data collection

The data for the study were obtained from (1) the analysis of the project documents and the strategic management documents of the selected HHCDs, followed by (2) the individual interviews with the staff members playing key roles in the development of the devices. The employment of multiple data sources was to gain a deep and holistic understanding of the diverse influential factors upon decision-making within HHCD development with different dimensions and realities, and continuously validate the findings produced during the research procedures (see Table 2).

**Table 2:** The collection and analysis of the data

Data sources	Analysis	Comments/Outcome
Stage 1: Analysis of the project documents and the strategic management documents of the selected HHCDs	Thematic, Statistical	Thirty-four open codes Nine initial themes to be further explored, e.g., insufficient understanding of the users, excessively use of second-hand user information
Stage 2: Interviews with the twelve project leaders	Thematic, Statistical	Forty-five open codes Four refined themes, i.e., source of user information, impact of user insights, factors determining the success of new HHCD development, reasons for developing new HHCDs Descriptive statistics, e.g., the frequency of the execution of in-house user research during the projects, the sources of user data applied in individual projects, and the critical reasons for developing new devices
Stage 3: Interviews with the six departmental representatives (besides the project leaders)		

In the first stage, we analysed the selected HHCDs’ project documents, including project proposals, design/product specifications, reports, and meeting records, along with the manufactures’ strategic management documents, including the principal new product development process and its supporting documents, business portfolio, and organisational structure charts. The results led to the generation of the initial codes, which then became themes and questions that needed to be further explored.

In the second stage, two individual interviews were conducted by the first author with each of the twelve project leaders (project/product managers) from M1-M5, who were the key informants (duration = 1–1.5 h each, sum≈25 h).

In the third stage, the first author interviewed M1′s six other departmental representatives (see Sect. 2.1), to both validate and to complement the information provided by the managers (duration = 1–1.5 h each, sum≈10 h). Before conducting each interview, we explained the purpose and format of the study to all participants. We also collected informed consents to participate and for the audio recording of the interviews.

The interview format was qualitative and semi-structured [31, 47] to reflect the exploratory nature of the study. Before conducting the interviews, a preliminary interview guide was prepared, which evolved as individual interviews progressed, allowing the interviewees to provide greater depth on matters that we found important [21, 32]. Exemplar questions included “what do you think are the factors determining the success of new HHCDs?”; “how do you make sure that the users’ real needs and requirements are properly considered in a project?”; “what are the sources of the user information?” (See Additional file 1 for the complete list of questions). We also collected quantitative data regarding the counts of events (e.g. The number of projects where formal activities of collecting user information were executed?), and project documents (e.g. how many projects have user input from the clients?). All interviews were audio-recorded and later transcribed.

To make sure the obtained data was authentic, original and reliable, the technique of member check was used during the interviews [10, 15]. More specifically, the first author restated and/or summarised a respondent’s answers and then asked him/her to determine the accuracy and avoid misunderstanding. He/she was then asked to carefully read through the transcripts, to either affirm or refute the authors’ interpretation of the data, upon the conclusion of the interviews. Only the confirmed information was used for further analysis.

All of the preceding activities took place at the company partner, either in a meeting room or at the informants' offices, if available. The data were transcribed and managed using NVivo (QSR International, Cambridge, MA).

### Data analysis and establishing rigour

**Fig. 1:**
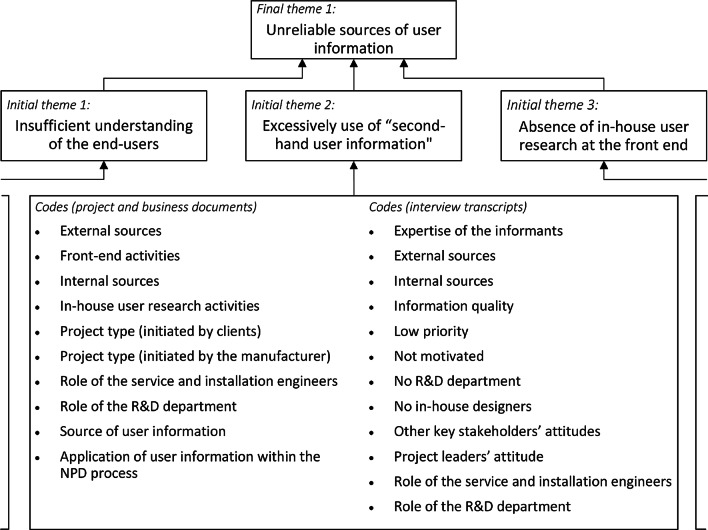
An extract from the code system

Given that a thematic analysis facilitates the effective and rigorous abstraction of salient themes and sub-themes from a complex and detailed textual dataset [14], the data in this study were analysed thematically [6]. Firstly, the obtained data were read multiple times by the authors to obtain a general sense of their natures. Secondly, open codes [48] were generated by labelling the essence or key attributes of the data. “Open codes” refer to the codes produced by open coding that is the initial phase of the coding process in the grounded theory approach to qualitative research [22]. Later on, through tentatively collating similar codes using affinity diagram [4], twelve initial themes such as “insufficient understanding of the users”, “excessively use of “second-hand user information”, and “absence of in-house user research at the front end” emerged. Altogether seventy-nine open codes (including sub-codes) were generated. Figure 1 outlines an extract from this code system (see Fig. 1). Initial themes were subsequently refined until a clear consensus of the final themes between the first and second authors was reached. The four final themes are: “sources of user information”, “impact of user insights on the NPD process”, “factors determining the success of new HHCD development”, and “reasons for developing new HHCDs” (see Table 2).

To guard against the potential for lone researcher bias, and also to acquire a rich description of the themes and theory development, peer review [35] was opted during the data analysis procedure. At the start, the first and second authors analysed both 20% of the project documents and 20% of the interview transcripts independently. Afterwards, they compared their findings and agreed on the codes and themes before the analysis proceeded. The Cohen’s Kappa value of agreement between the two coders was 0.69 on coding the project documents, and 0.74 on coding the interviews, indicating a “substantial level” of the agreement [33]. The first author then analysed the rest of the data. Descriptive statistics were reported for the quantitative data collected in this study.

## Results

### Sources of user information

Both the interviews with the informants and the analysis of the project documents showed that the user insights utilised in the development of the thirty-eight selected HHCDs were established from the four sources: clients, team members’ intuition and experience, service and installation practices, and in-house user research. The clients of the devices were mainly the public sector, including local authorities and housing associations, and non-hospital healthcare facilities.

As presented in Table 3, intuition and experience influenced team members’ user insights that were utilised within the development of all of the HHCDs investigated; and was the sole source of user knowledge for 18.4% (n = 7) of the devices. In addition to intuition and experience, user information for 28.9% (n = 11) of the devices came solely from clients; 21.1% (n = 8) came solely from service and installation practices; 2.6% (n = 1) came from a combination of clients and in-house user research, and 5.3% (n = 2) came from a combination of in-house front-end user research, and service and installation practices. For 2.6% (n = 1) of the devices, user information came from all of the four sources. Overall, the clients contributed to 55.3% (n = 21) of the devices with user information; service and installation practices to 50% (n = 19) of the devices; and in-house front-end user research to only 10.5% (n = 4) of the devices (see Table 4).

**Table 3:** Sources of user information utilised in the projects investigated

Sources of user information				Number of HHCDs (sum = 38)
Clients (the public sector)	In-house front-end user research	Service and installation practices	Team members’ intuition and experience	
×			×	n = 11
×	×		×	n = 1
×		×	×	n = 8
×	×	×	×	n = 1
	×	×	×	n = 2
		×	×	n = 8
			×	n = 7

**Table 4:** The number of HHCDs to which respective sources of user information contributed

	Sources of user information	Number of HHCDs (sum = 38)
In-house sources	In-house front-end user research	n = 4 (10.5%)
	Service and installation practices	n = 19 (50%)
	Team members’ intuition and experience	n = 38 (100%)
External sources	Clients (The public sector)	n = 21 (55.3%)

Fifty-eight per cent (n = 22) of the selected devices were initiated, reflecting the direct requests and the conceptual product ideas from the clients. In the project briefing documents of 90.9% (n = 20) of these projects, the clients described their own insights of the target users. In 70% (n = 14), these insights were associated with field data collected by the clients. The user information from the clients was considered effective and credible by all of the key informants (product managers and project managers, n = 12). These project leaders even took the initiative to request user information from the clients, in 18.8% (n = 3) of the projects where project initiators were the managers themselves.

In terms of internal information sources, service, and installation practices contributed to the development of 50% (n = 19) of the devices with user information. This was the sole information in 5.2% (n = 2) of the cases, besides team members’ intuition that was based on their experiences from the past work they were involved in or other projects/devices that they were aware of. Service and installation practices were regarded by all of the five companies in this study as an economical and efficient in-house source of user information. For example, as per our conversation with M3′s project manager:

The users were only engaged during the testing phase of these devices… All of the user tests were carried out by the installation engineers.

The most common reason extracted from the interview results was that the service and installation engineers interacted with the end-users in everyday work, e.g., to solve technical problems, install new products and replace components. The other important reason was that there was no formal Research and Design (R&D) department/team to undertake user research concerning M2-M5. However, both of the two interviewed service and installation engineers pointed out that studying users was above and beyond their duties, nor were they trained or received full support for doing this work.

As for M1, its R&D department neither led nor performed user studies, in the majority of the cases. Its workload was actually concentrated on reducing manufacturing costs, carried out under the operational director’s leadership. For example, the R&D manager said:

Our team works mainly to meet the constant new requests from the operational director.

This was inconsistent with the quality director’s description, which indicated that the R&D department should report to both the product managers and to the operational director, in everyday NPD practices. As per the innovation director:

The workload of the R&D department should be shifted from supporting the manufacturing and documenting to design-related activities.

### Impact of user insights on the NPD process

All of the five manufacturers in this study had forged their own principal/global NPD processes. For example, the medium-sized manufacturer employed a staged/waterfall process [9] comprising of six major phases (i.e., discovery, scoping, building the business case, development, testing & validation, and launch), and over eighty secondary stages and activities. A principal NPD process is meant to be referred to by all projects of a company. It serves as the “bible” for guiding every day NPD practices, as described by an interviewed innovation director.

We found that only M1 and M4′s principal NPD processes incorporated pre-planned activities relevant to the production and application of user information. All of these activities were positioned in the later stages, when device designing had been completed, without exception. Users would only be engaged during the tests of the Alpha, Beta and/or pre-release versions of a new device. The purposes of the tests, as we summarised from analysing the project documents, included: ensuring a selected design meet business requirements and design specifications before mass production rolls out; providing essential user feedback as required by standards and policies in the sector; and facilitating new device launch by demonstrating its outstanding usability, performance and functionality. The evidence of the user research associated with strategy formulation or device designing, in any format, was present in the project documents of only 10.5% (n = 4) of the investigated devices.

From comparing the answers given by the different interviewees, we found that the structures of the NPD processes had led to inconsistency among team members’ perception of engaging the end-users and of adopting formal UCD methods. For example, one senior product manager from M1 pointed out that:

… user information from the service and field engineers could hardly fit into the current NPD process.

And a service engineer from the same company:

Frustration occurs as I am in the middle of user views and company strategies.

This issue reduced some staff members’ willingness to collect and transfer user information. As per a senior installation engineer:

Even when I have fed back to the company design ideas or suggestions from the users, I often do not know what has happened to them. It is like a black hole.

### Factors determining the success of new HHCD development

**Fig. 2:**
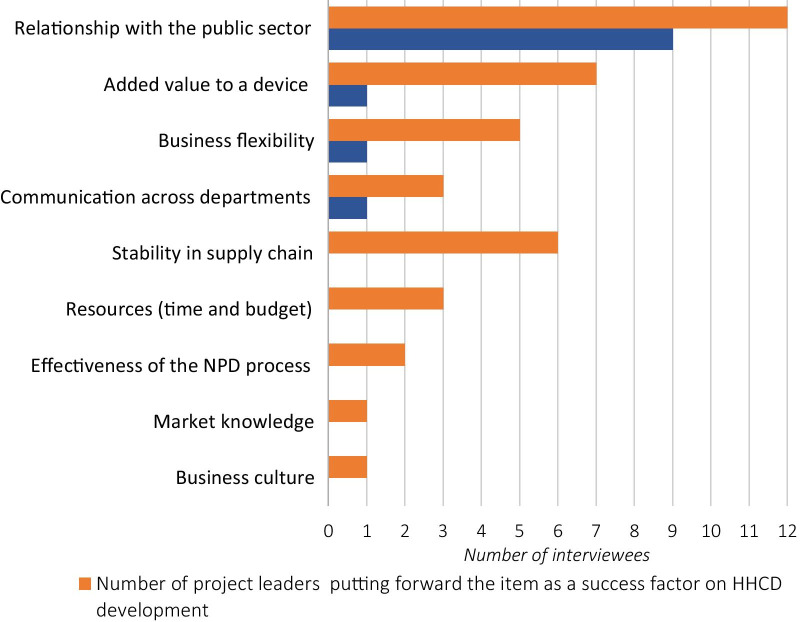
The nine critical factors influencing the success of HHCD development

The interviews with the project leaders indicated nine essential factors that influenced the success of new HHCD development: (1) relationship with the public sector, (2) added value to a device, (3) business flexibility, (4) communication across departments, (5) stability in the supply chain, (6) resources (time and budget), (7) effectiveness of the NPD process, (8) market knowledge, and (9) business culture (see Fig. 2). Among these items, a manufacturer’s relationship with the public sector was put forward by all of the project leaders interviewed in Stage 1 as the most important success factor; and was regarded by 75% (9 out of 12) of them as the most influential factor. For example, M1′s product manager indicated:

Our strong long-term cooperation with the purchasing organisations was the biggest advantage over the competitors.

The other three project leaders considered added value to a device, business flexibility, and communication across departments as the most influential factors.

In addition to the above factors, effective user knowledge was considered essential to the success of new HHCD development, by the R&D manager, the service manager, and the innovation director who were interviewed in Stage 2 of the research. However, effective user knowledge was not regarded by any of the three interviewees as the most influential success factors.

### Reasons for developing new HHCDs

The analysis of the project documents (i.e., the selected HHCDs) identified three main reasons from which new HHCDs were derived. These reasons included: client request for a new device (57.9%, n = 22), the manufacturer’s own business strategy (31.6%, n = 12), and changes to legislation and laws (10.5%, n = 4). There was no trace of a device initiated reflecting the findings in terms of the end-user.

The above finding was consistent with the results from the interviews with the twelve project/product managers, which affirmed that the client request was the most common and critical reason for initiating the development of a new HHCD—this opinion was expressed and agreed by 91.7% (n = 11) of the respondents. According to them, the primary clients for 71.1% (n = 27) of the devices were public organisations, including local authorities and housing associations, and non-hospital healthcare facilities. These organisations purchased the devices and then provided the devices to the residents who needed them. For example, during the interview M4′s project manager said:

XXX (the model name of a telecare home unit) had been installed in each bungalow (council house) of the community before the residents moved in.

## Discussion

Our results revealed that the design of most (89.5%, n = 34) HHCDs in this study were based on insufficient user insights. This owed to four main reasons:

Firstly, user insights utilised in formulating project strategies were established solely from team members’ intuition and experience, for 18.4% of the HHCDs in this study (see Sect. 3.1). Exploiting intuition in decision-making at the front end of NPD increases new product creativity [12], whereas making intuitive judgments alone may lead to inaccurate or erroneous decisions [20]. Although our results presented in Sect. 3.1 showed that there was unanimity among those interviewed that intuition was strongly related to “the right experience and knowledge”, it has been widely acknowledged that intuition needs to be used in addition to generally accepted rational approaches, in fuzzy front-end decision-making (e.g., [3, 13, 16]. Even for a combination of intuitive and cognitive judgment, its effectiveness can be associated with other factors, for example, the level of stress under which NPD teams are working [12].

Secondly, the manufacturers investigated tended to depend on the “second-hand user information” which quality cannot be promised. As elaborated in Sect. 3.1, the public sector including local authorities, housing associations, and non-hospital healthcare facilities contributed to 44.7% (n = 17) of the projects investigated with user information, which was more than any other user information sources determined in this study. User information acquired from these organisations can be beneficial, given the information providers’ expertise in the medical and healthcare area and their high frequency of engaging HHCD users in every-day work. However, the credibility, accuracy, completeness and broadness of the information cannot be promised, particularly from the new device design perspective. This is because that the activities of information collection were undertaken by those from external organisations, at different locations, using unknown methods, and with purposes that might not be in line with the strategy and/or requirements of a specific project. In 28.9% (n = 11) of the cases investigated, the public sector was the only source of user information, apart from the team members’ intuition and experience (see Sect. 3.1).

Thirdly, there was a lack of formal user research conducted by NPD teams, and to support the product design processes, in the majority (89.5%) of the cases. Not only the new entrants and mid-tier companies, namely M2- M5, the incumbent HHCD provider, namely M1, was no different. Even with a formal R&D department, target users “were only engaged during the testing phase” of their NPD processes, as pointed out by the staff members of the company (see Sect. 3.1). As a result, service and installation practices became the internal source of front-end user information. They contributed to five times the investigated cases of which front-end user information came from in-house user research, as set out in Table 2. Despite the benefit of producing quantitative data at relatively low cost, this approach of acquiring user information has been found in this study to have serious drawbacks. On the one hand, producing accurate, rich, latent and unarticulated user information, as needed for formulating NPD strategies and designing new devices, was not the purpose of the activities where the information came from. On the other hand, producing effective user information was above and beyond the duty of service and installation engineers, nor were they adequately equipped in doing this task.

Fourthly, the manufacturers’ principal NPD processes failed to provide the necessary guidance and support for the establishment and enforcement of rich and valid user insights. The design of a principal NPD process can influence every project at a company by providing company-wide criteria for NPD [53]. As set out in Sect. 3.2, none of the five manufacturers’ principal NPD processes reflected UCD principles—only two of them determined when and how to engage users; and the user engagement had little to do with strategy formulation and new device designing. This fact contributed to the absence of user engagement at the front stage that however is widely acknowledged to be the best opportunity to influence the end device (e.g., [2, 13, 29]. Additionally, without being defined and clarified by a principal NPD process, team members’ understanding of the roles of the end-users tended to be inconsistent. This resulted in hesitation and frustration in producing and utilizing user information, from both the information producers and the consumers.

The unsatisfactory situation of obtaining and applying user information was primarily attributed to our finding that the manufacturers were unwilling to deploy formal UCD/human factors methods or to engage in structured research activities exploring the needs and features of the end-users. The most critical cause was that most HHCD development projects were initiated following the requests from the public sector, and the end-users were perceived as having little impact on the project success. This confirms and further extends the research of Van Kuijk et al. [51] that concludes “whether developers think that usability is a purchase consideration for their clients seem to influence the prioritization of usability”. As set out in section “Reasons for developing new HHCDs”, the reasons for initiating new HHCD development projects can be divided into three types, i.e., client request, the manufacturer’s own business strategy, and changes to legislations and laws. Client request was most common and critical, as concluded from both the analysis of the project documents of the selected devices and the interviews with the project leaders (product/project managers). Unlike many other consumer products like cell phones or vehicles, in the HHCD sector, the major clients/buyers and the end-users are often two different groups of people. The direct clients for most (71.1%, n = 27) of the selected devices were the public sector, although the end-users might also be charged by the manufacturers for device associated services such as maintenance, health monitoring, training and other follow-up support.

The interviewed project leaders indicated nine essential factors determining the success of HHCD development, whereas none were related to the end-users (see section “Factors determining the success of new HHCD development”). These factors can be categorised into two groups: serving sale purposes (i.e., relation with the public sector, added value to a device, stability in the supply chain, and market knowledge); and serving project management purposes (i.e., business flexibility, communication across departments, resources, and business culture). These success factors affirmed Money et al.’s [39] argument that the medical device developers had a strong sales focus, “seeking device design input from those individuals who make purchasing decisions, as opposed to the users of the devices”. Although some interviews representing other functional groups (i.e., the R&D manager, the service manager, and the innovation director) considered effective user knowledge to be an essential success factor, these interviewees had little influence relative to the project leaders on the progress of a project.

Obviously, the manufacturers’ criteria for the success of new HHCDs was different from that of the academics and practitioners (e.g., [5, 7, 34, 44] who argued that the designing of HHCD must focus on user factors and experience. This partly explained why few front-end user research activities were found during the review of the project documents. Employing formal human factor engineering/UCD methods in healthcare device development has been said to bring a number of benefits related to health outcomes and commercial success. For example, improved user satisfaction and safety by ensuring appropriate consideration to the users’ work patterns, and their individual needs, and the environment in which the device is to be used [46],and substantially reduced device development time by avoiding costly design changes and product recalls [11]. For focusing on the end-users to become an organisational priority, it needs support from a range of actors that build a formal and informal network around the idea (Kijkuit & Van Den Ende, 2007; [37].

### Limitations of this study

Due to time and budget limitation, a relatively small sample of HHCDs was analysed in this study. Although the research targets incorporated many of the major types of HHCDs on the market, some types were still excluded, for example, first aid equipment, infant care equipment, and treatment and therapy equipment. The development processes of these devices may have some characteristics that have not been addressed in this study.

Additionally, we could only access the project leaders and the project information of three devices produced by M2-M4, respectively. To investigate an extensive portfolio of HHCDs and to access the staff members from different company divisions, the first author provided design and consultancy services over a long-term window. This could not be repeated in the other four companies. While the project leaders are most critical in providing the holistic project information of the selected devices, other stakeholders participating in the NPD processes can also contribute to this study with valuable project information.

## Conclusions

Our previous article [55] indicated that the designs of many HHCDs do not reflect all of the critical needs and requirements of the end-users. This study further explored how user insights were obtained and utilised in designing HHCDs and indicated that the manufacturers’ NPD approaches failed to reflect the principles and requirements of human factors methods. The four critical reasons for the application of insufficient user insights included: (1) the user insights utilised in formulating project strategies and the development of new devices was established solely from team members’ intuition and experience; (2) the HHCD development teams were dependent on the “second-hand user information” provided by the public sector; (3) the formal user research conducted by NPD teams was absent; and (4) the manufacturers’ principal NPD processes failed to provide necessary guidance and support for the establishment and enforcement of rich and valid user insights.

The issues above could be partly attributed to the manufacturers’ low commitment and motivation in deploying formal user research, that existed in all of the manufacturers in this study. One root cause was that most of the HHCDs were initiated following the requests from the public sector (the clients), and the end-users were considered to have little impact on the success of the projects. This suggests that the integration of user insights in the development of HHCDs may not be improved to a significant level without changes to the current business model, which would require deliberate efforts from the purchasing organisations, as well as further amendments to some current standards and regulations in the sector. There is a need for a legal requirement that can force effective enforcement of human factor engineering/UCD methods within the development of HHCDs. On the other hand, business feasibility must be adequately taken into consideration in the establishment of future methodologies and guidelines, given the gulf between the wide recognition of UCD principles and the unsatisfactory adoption of the principles in HHCD development practices.

## Supplementary Information

**Additional file 1**. Theinterview questions.

## Data Availability

All data generated or analysed during this study are included in this published article.

## Abbreviations

HHCD: Home healthcare device
NPD: New product development
SME: Small and medium-sized enterprise
UCD: User-centred design
