# Supplementary material for: User knowledge factors that hinder the design of new home healthcare devices: investigating thirty-eight devices and their manufacturers
Source: BMC Med Inform Decis Mak. 2021 May 21;21:166. doi: 10.1186/s12911-021-01464-3 (PMC8139000; doi:10.1186/s12911-021-01464-3)
Supplement: Supplementary file 1 — Additional file 1. Theinterview questions. [file 12911_2021_1464_MOESM1_ESM.docx]

**Additional file 1. The interview questions**

1. What do you think are the key factors determining the success of a new HHCD?
2. Please list the main reasons of initiating new HHCDs in the projects that you were involved.
3. Please use a few sentences to describe the role of the users within the development of a new HHCD.
4. In your projects, how do you make sure that the end-devices meet the real needs and wants of the users?

***The users of the selected devices***

1. Who are the users of the device?
2. Are the users also the customers/buyers of the device? If not, who are the buyers/customers?
3. Are the actual users fully consistent with the intended users during the NPD process? If not, what are the reasons for the engagement of new users?
4. Are the actual customers/buyers fully consistent with the intended customers/buyers during the NPD process? If not, what are the reasons for the engagement of new customers/buyers?
5. Could you please describe the relation between the users, the customers and the manufacture (your company)?

***The NPD processes***

1. What were the reasons of developing this device?
2. Please describe the NPD process applied during the development the device.
3. Who created this process?
4. Were there any user research activities performed during the process?
5. If yes, please describe the activity/activities, and who performed the activity/activities.
6. If yes, did the activities’ outputs justified the resources that the activities consumed?
7. If yes, were there any other sources of user information?
8. If not, where did the user information applied in the project come from?
9. Which source of user information contributed most to the creation of the end-device?
10. Are the sources of the user information reliable?
11. Is there a principal NPD process at your company that every NPD project need to comply with?
12. If yes, what are the strengths and weaknesses of the principle NPD process?
13. If yes, is the principle NPD process applied in the development of all of the three devises? If not, what are the causes?
14. If yes, do you always follow the principle process in your projects? If not, why?
15. If yes, does this process incorporate any pre-planned activities relevant to the production and application of the user information?
16. If yes, is this process effective in acquiring, processing, delivering and utilising user information? (details required)
17. If not, are there any major differences between the NPD processes of the three devices? If yes, what are the differences?
18. If not, how did you select/forge the NPD processes as required by different projects, to make sure that the end-device properly reflect the real needs and wants of the users?
